# Two interventions to treat pain disorders and post-traumatic symptoms among Syrian refugees: protocol for a randomized controlled trial

**DOI:** 10.1186/s13063-019-3919-x

**Published:** 2019-12-27

**Authors:** Wegdan Hasha, Lars T. Fadnes, Jannicke Igland, Rolf Vårdal, Line Merete Giusti, Elisabeth Marie Strømme, Jasmin Haj-Younes, Unni Heltne, Bernadette N. Kumar, Esperanza Diaz

**Affiliations:** 1 Department of Global Public Health and Primary Care, University of Bergen, Bergen, Norway; 2 Department of Addiction Medicine, Haukeland University Hospital, Bergen, Norway; 3 Center for Migration Health, Bergen, Norway; 4 Centre for Crisis Psychology, University of Bergen, Bergen, Norway; 5 Unit for Migration and Health, Norwegian Institute of Public Health, Oslo, Norway

**Keywords:** Randomized controlled trial, Mental health, Chronic pain, Teaching Recovery Techniques, Physiotherapy, Refugee

## Abstract

**Background:**

There is a high prevalence of pain and post-traumatic symptoms among refugees and feasible interventions to manage these are needed. However, knowledge about the effect of physiotherapy and psychological group interventions among refugees is scarce. Our aim is to determine whether two different interventions, the Physiotherapy Activity and Awareness Intervention (PAAI) and Teaching Recovery Techniques (TRT), reduce pain and post-traumatic symptoms among refugees from Syria living in Norway.

**Methods/design:**

Syrian adults with either pain disorders or post-traumatic symptoms, or both, will be recruited to this randomized control trial. The trial will include two separate interventions: participants with dominating pain symptoms will be assigned to the PAAI; and those with a predominance of post-traumatic symptoms will be assigned to the TRT intervention. Participants will be randomized to either the immediate intervention group or the delayed intervention group, for each of the interventions (PAAI and TRT). A minimum of 68 participants will be recruited for the PAAI and 78 participants for TRT, in order to detect clinically and statistically significant symptom improvement, assuming 25–30% attrition after recruitment. The main outcomes for the analyses will be pain intensity measured by the Brief Pain Inventory questionnaire and the scores of the Impact of Events Scale — Revised. The effect will be evaluated at the end of interventions lasting 8 weeks (PAAI) and 6 weeks (TRT) using the same instruments after the end of the intervention, and again 4–6 weeks later. Additionally, a qualitative evaluation will be conducted through an embedded process evaluation and personal interviews with participants after each of the interventions is finished.

**Discussion:**

Our study will determine the feasibility of the implementation of two different interventions and the effect of these interventions among refugees from Syria with pain disorders and/or post-traumatic symptoms.

**Trial registration:**

Clinical Trials.gov, NCT03951909. Retrospectively registered on 19 February 2019.

## Background and rationale

Both chronic pain and mental health symptoms can be a consequence of traumatic events [1, 2]. At the beginning of 2019, according to the United Nations High Commissioner for Refugees (UNHCR), 70.8 million displaced persons due to war and violent conflict were registered worldwide, of which 25.9 million were refugees. Syrians are one of the growing groups that have fled to Europe since the war began in 2011 [3, 4]. Norway is no exception, with more than 30,000 Syrian refugees living in the country by the end of 2018 [5–8]. Many refugees have been exposed to stressful events or situations that can lead to persistent distress [9]. As a consequence, the presence of pain disorders and mental health symptoms is common, both of them often in combination within the same individual [10, 11].

The complex relationship between torture, pain and other aspects of the individuals’ experience before and after migration has profound impacts on the everyday lives of many refugees [12]. Pain has been identified as a predictor of emotional distress among refugees [13], but there is a scarcity of population-based data about pain disorders among refugees from Syria. According to a Norwegian study, 76% of the traumatized refugees attending an outpatient clinic experienced chronic pain [14].

Mental problems of various types and degrees are prevalent among refugees, including Syrians. In Lebanon during 2011–2013, 44% and 61% of Syrian refugees reported depression, and some of them also loneliness, in two similar studies [15, 16]. In 2014, 54% of Syrians accessing International Medical Corps facilities in Syria and neighboring countries suffered severe emotional disorders, including depression and anxiety [9]. Among the Syrian refugees resettled in the USA, a high prevalence of post-traumatic stress disorders (PTSD) (32%), anxiety (40%) and depression (48%) has been reported [17].

Health care services for refugees during flight and in host countries shortly upon arrival are either non-existent, inadequate or insufficiently available, especially regarding chronic pain and mental health problems [18]. Inadequate health care for refugees can worsen their symptoms and lead to chronicity [10]. Once established in a host country, the national health services in most high-income countries are obliged to offer health care services to refugees. However, the lack of evidence regarding the best treatment choices for this population, together with limited resources, may compromise the health services provided to refugees. In addition, treatments are often only entitled to those with formally established diagnoses. However, many refugees who do not have enough symptoms for a formal PTSD or chronic pain diagnosis might still have symptoms like headaches or flash backs that go unmanaged and impede their learning of a new language and integration into society. It has been suggested that group treatment could be an approach to maximize the effect of the treatment for coping with symptoms among traumatized refugees, as well as a means to increase social interaction and well-being among participants [19].

The evidence on group-based physiotherapy for chronic pain among refugees is scarce. As far as we know, only a study in progress in Denmark has used mixed physical activity and basic body awareness therapy to reduce chronic pain for refugees with established PTSD [20]. A combination of psychomotor and general physiotherapy exercises is often used in Norway and other Nordic countries in group therapy, as this enables the health professional to recognize and normalize the participants’ emotions and movement patterns, capturing and preserving the individual treatment needs within the group, at the same time as having an overview of the social interactions [21]. However, the adequacy or effect of this therapy has not been well studied for refugees. The Physiotherapy Activity and Awareness Intervention (PAAI) package was adapted for this study based on this combination of psychomotor and general physiotherapy, and grounded on the extensive experience with immigrant patients of the physiotherapists in the team and according to users’ recommendations.

Teaching Recovery Techniques (TRT) is a self-help group intervention designed and evaluated for children by the Children and War Foundation for use on subjects exposed to war or natural disasters [22]. TRT is built to meet the needs of a large number of traumatized refugees who require interventions for mental support. It is based on the principles of cognitive behavioral therapy and on evidence-based methods for treating trauma. The intervention focuses on three main groups of symptoms following severe traumatization, namely invasive sensory impressions, bodily activation and avoidance. TRT has shown significant effect in terms of reducing the aforementioned symptoms among children surviving catastrophes and adolescent asylum seekers in Europe [23]. Among adolescents in Palestine, TRT significantly reduced post-traumatic stress symptoms and was evaluated as a cost-effective intervention [24]. However, the effect of TRT on adult refugees with post-traumatic symptoms has not been studied.

Taking the previous into consideration, there is a need to develop and evaluate techniques and methods to adequately deal with adult refugees with chronic pain and post-traumatic symptoms.

### Objectives

The main objective of this project is to evaluate the effect of two different interventions, separately, among adult Syrian refugees with pain and/or post-traumatic symptoms:

- The Physiotherapy Activity and Awareness Intervention (PAAI) in reducing the degree of pain, and secondarily in reducing post-traumatic symptoms (if present)
- The Teaching Recovery Technique (TRT) group intervention in reducing the degree of post-traumatic symptoms, and secondarily in reducing the degree of pain (if present)

## Methods

### Study design

**Fig. 1:**
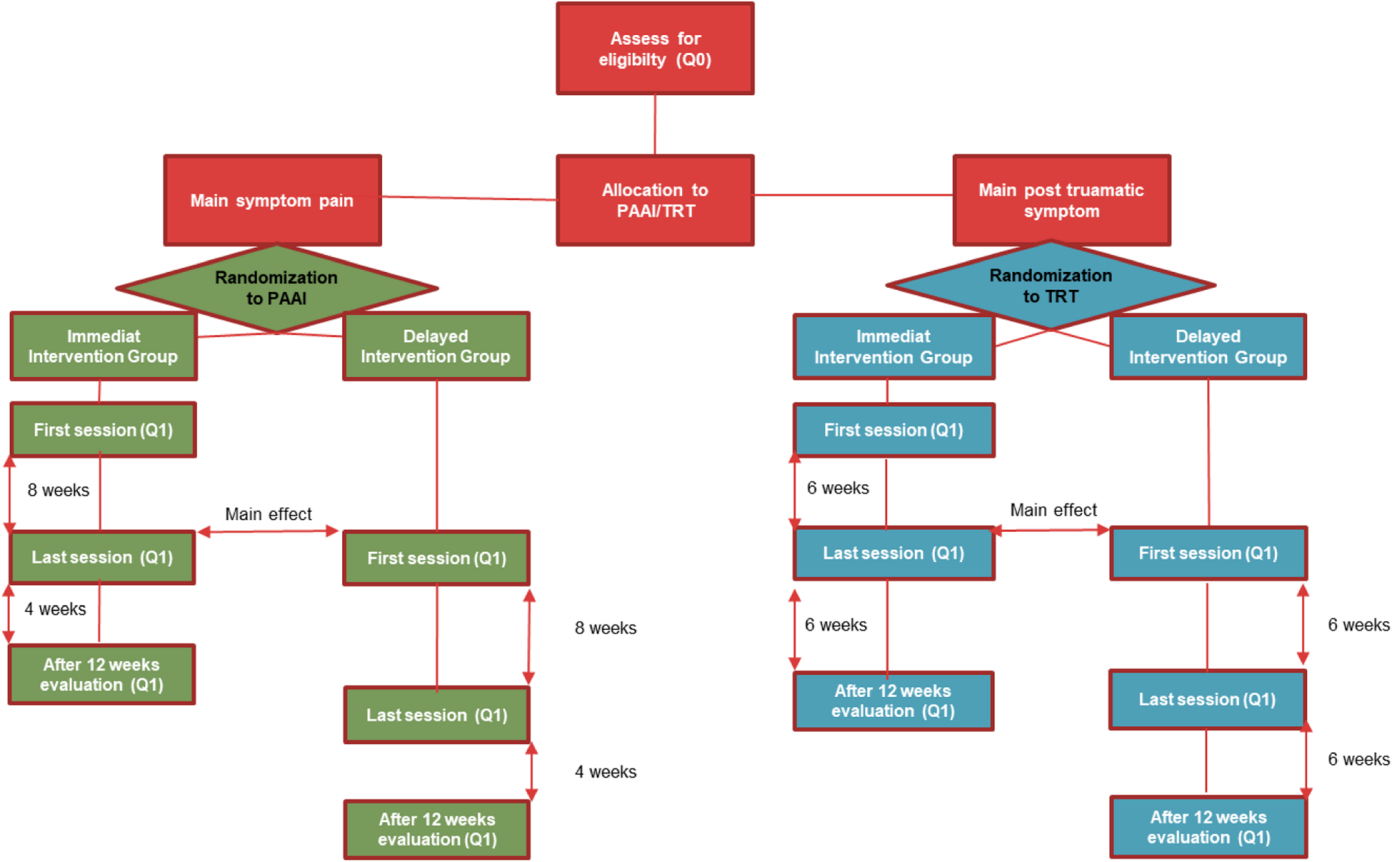
Flowchart overview of the interventions. PAAI Physiotherapy Activity and Awareness Intervention, Q0 Questionnaire 0, Q1 Questionnaire 1, TRT Teaching Recovery Techniques

**Fig. 2:**
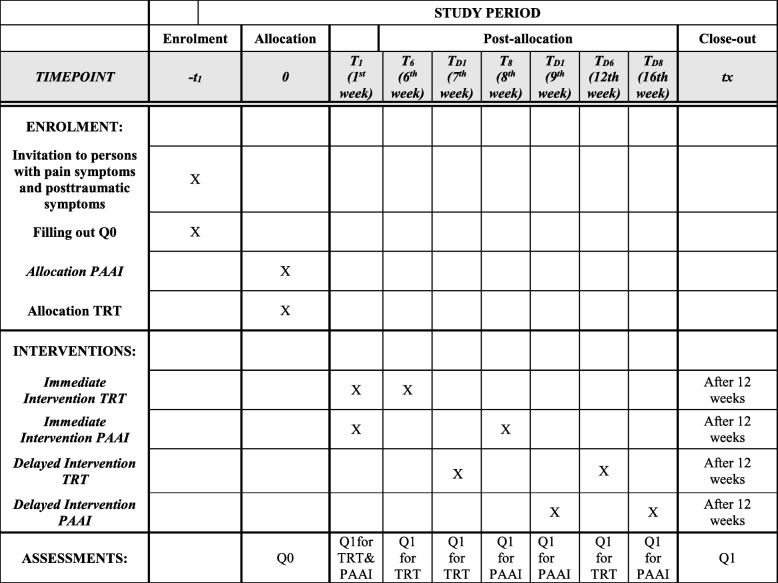
Standard Protocol Items: Recommendations for Interventional Trials (SPIRIT) schedule of enrolment, interventions and assessments. PAAI Physiotherapy Activity and Awareness Intervention, Q0 Questionnaire 0, Q1 Questionnaire 1, TD delayed intervention (same intervention at a later time point), TRT Teaching Recovery Techniques

This is a 2 × 2 armed randomized control trial (RCT) to study the effect of two different interventions for treating pain and post-traumatic symptoms (Fig. 1). Participants with dominating pain symptoms will be assigned to the PAAI and those with a predominance of post-traumatic symptoms will be assigned to the TRT intervention. Participants within each of the arms are randomized to either the immediate intervention group or the delayed intervention group, which will receive the same intervention but at a later time point. The study protocol follows the SPIRIT recommendations for intervention trials (Fig. 2, Additional file 5).

In addition, to examine the process of how the interventions improve health, a qualitative embedded process evaluation using personal observation of the intervention groups and individual semi-structured interviews will be conducted to gain an in-depth understanding of the mechanisms of action and key contextual variables affecting the intervention.

### Study setting

People suffering from chronic pain in Norway are generally followed up by primary health care including general practitioners and physiotherapists. However, in many regions including Bergen there is limited availability of treatment and follow-up, especially when an interpreter is needed. Similarly, people suffering from mental health suffering are generally followed up by primary health care and/or tertiary health care including general practitioners and psychologists (generally with need for referral). However, in many regions including Bergen there is limited availability of follow-up by psychologists, and specific cultural competence is scarce.

### Participants and recruitment

We recruit adult Syrian refugees (age ≥ 16 years) living in Bergen and adjacent municipalities from several arenas: educational activities in which the newly arrived immigrants initially are enrolled in groups of approximately 30 students; patients from health care providers both in primary and secondary care and pharmacies, especially from areas with a high number of immigrants; and other non-health related locations where refugees often gather, like specific immigrant shops (e.g. Middle-Eastern and Asian grocery stores). In these settings, adults from Syria are shortly informed about the study in Arabic by the first author, who speaks Arabic and Norwegian. Adults with either pain or post-traumatic symptoms who are willing to participate are given an individual appointment for further information and potential inclusion. At this point, those consenting fill out the baseline questionnaire (Q0 — see Additional file 1). This questionnaire is used to identify participants fulfilling the inclusion criteria regarding pain and/or post-traumatic symptoms. All participants are invited to contact the first author for more information at any point of the study.

### Inclusion criteria

Participants have to report either pain or post-traumatic symptoms in order to be included in one of the two interventions. Patients with physical pain are included if they report chronic pain that has lasted more than 6 months and score 3 or higher for either of the two items about pain severity on the Brief Pain Inventory (BPI) short questionnaire assessing average and current pain ranging from 0 to 10 [25]. Patients who answer yes to the question “Have you experienced any of these or some other terrifying event(s)?” and score over 24 on the revised Impact of Events Scale (R-IES) are included. The IES-R yields a total score ranging from 0 to 88 [26]. Patients scoring 20 or more on the General Health Questionnaire-12 (GHQ-12) are referred to a consultation with one of our collaborating psychologists in Bergen municipality before invitation to group participation, to make sure that he or she can benefit from group participation. If group participation is considered unfitting, the patient is referred to individual treatment.

Participants are allocated to either the PAAI or the TRT intervention according to their symptoms. Participants who present both pain complaints and post-traumatic symptoms in whom symptom scores measured as a relative percentage of the maximum respective scales, BPI and IES-R, are highest are invited to the intervention treatment.

### Exclusion criteria

Potential participants are excluded if they report health conditions requiring close medical follow-up, like diabetes with complications or cancer under treatment, or score on the mental health questions as having a serious mental illness (25 or more on the GHQ-12) and are assessed by psychologists to need individualized therapy. These patients will be referred to the appropriate level of care. Practical situations that impede attendance to treatment on a regular basis, like living far away from the therapy locations, are also a reason for exclusion.

### Allocation and blinding

A random allocation sequence was generated by a statistician using the ralloc command in Stata version 15. The sequence was obtained using a 1:1 allocation ratio in block randomization with block sizes varying between 4, 6 and 8. Separate randomization sequences were made for the PAAI and TRT. After generation of the lists, each line was numbered from 1 upward, reflecting the order of recruitment (participation number). Participants were individually randomized. To be able to recruit within periods that are shorter than the time delay between intervention and delayed list groups (6–8 weeks), and in order to be able to provide interventions of adequate size, recruitment is organized in three waves followed by three rounds of interventions.

It will not be possible to blind the participants or the instructors in this study during the active intervention phase. The person who recruits participants and assigns participation numbers does not have access to the randomization list. Unfortunately, this person (WH) is both recruiting and assessing outcomes and we only have one statistician, so further blinding is not possible.

### Interventions

In collaboration with Bergen municipality, the Centre for Crisis Psychology and the users, two interventions were developed and adapted to our population.

#### Physiotherapy Activity and Awareness Intervention (PAAI)

The physiotherapy treatment is based on principles from Norwegian psychomotor physiotherapy and general physiotherapy exercises and is led by physiotherapists working at Bergen municipality. In order to ensure the follow-up and guiding of each person, and to be able to handle reactions that might occur, 10–12 persons are invited into each group. Interpreters are oriented on their role in the group as well as the exercises. The words and phrases the therapists will use for instruction are shared with the interpreters beforehand, as there might be variants of Arabic depending on the country background of the interpreters. The intervention consists of eight sessions lasting approximately 1 h each with the same key elements each time: an introduction with welcome ball game and mindfulness exercises; sitting on the chair with a number of movements; lying down and relaxation; standing proprioceptive exercise; active movements stimulating balance, coordination and breathing; and, finally, grounding and short closing round.

The instructors explain the therapy and advise the participants to pay attention to their own limitations, for example, regarding pain and range of motion. Each participant is encouraged to do as much as possible of the exercises, within her or his capability at the beginning of each session. Otherwise, any injuries will be reported in the study and followed by the physiotherapists within the regular health care system.

#### Teaching Recovery Techniques (TRT)

TRT is designed in a step-by-step practical way to develop skills and techniques helpful in coping with the psychological effects of serious traumatization. In our case, the manual is adjusted for adults, with relevant examples and homework. All health professionals and collaborating interpreters involved in this intervention have previously been trained on the TRT manual by the Centre for Crisis Intervention. TRT is scheduled to one session weekly for 6 weeks; one session lasts approximately 2.5 h with up to 15 participants. The sessions deal with intrusive thoughts and feelings, arousal and avoidance in this order. To avoid secondary exposure and traumatization, participants are not required to disclose examples from their own life during group meetings, but they are asked to bring back memories of what happened to them. Participants will have tasks that are considered safe to carry out as homework.

### User involvement in development of the interventions

Representatives from the Syrian population in Bergen participated in a consulting group and contributed to the development of the interventions in May–June 2018. Eight persons from Syria, including both women and men, with different backgrounds and positions in society, were recruited and asked for their general opinion of the interventions regarding various subjects. These included: the need for gender-segregated or age-segregated groups; the frequency, the most appropriate time of the day and day of the week to conduct group sessions; the importance of socializing; the need to coordinate childcare during the sessions; the necessity of reminders (by SMS) prior to each session; giving recommendations on appropriate clothing for the sessions; preferences for interpreters; and so on. We will try to follow this advice as much as possible, including separate groups for women and men, in the implementation of the interventions.

### Sample size calculation

For calculation of statistical power, we assumed that differences at baseline will be random for the immediate intervention and delayed intervention groups. We calculated the necessary sample size using an independent-sample *t* test with 80% power and a significance level of 5%. We used mean and standard deviation values based on earlier findings by our research group among 150 refugees from Syria in Lebanon waiting to be sent to Norway (not yet published).

For the PAAI, we assumed a mean of 6.0 and SD of 3.8 in the BPI “normal” pain scale (range 0–10) and considered as clinically significant a difference of 3 points on the given scale. These calculations gave a minimum size of 27 participants in each of the immediate and delayed PAAI groups. Assuming 25% attrition, the numbers needed are 34 participants in each of the study arms; this is to say, a total of 68 participants are needed for the PAAI. Since each PAAI group will have approximately 10–12 participants, we will need 3–4 immediate intervention groups and 3–4 delayed intervention groups for the PAAI. Similarly, for the TRT intervention we assumed a mean of 35.6 points and SD of 15, 5 on the IES-R scale (range 0–80) and 13.1 points in change as clinically significant (and SD of 0.75). These calculations gave a study size of 30 participants for the immediate and delayed TRT groups. We allowed a 30% dropout for this type of intervention, giving 39 participants per study arm. Thus, 78 participants are needed for TRT. Since each group will have approximately 10–12 participants, we will need 3–4 immediate intervention groups and a similar number of delayed intervention groups for the TRT intervention.

### Measurements

Under the guidance of a bilingual field worker, two self-completed questionnaires in Arabic will be used. The more comprehensive baseline questionnaire (Q0) is used for the identification of participants as already described. A follow-up questionnaire (Q1 — see Additional file 2) will be used at the first and last sessions of the interventions as well as 12 weeks after the first session. The same questionnaires will be used for both interventions, TRT and the PAAI. The questionnaires have three parts covering: socioeconomic and migration-related information, which is more extensive in Q0; well-being and sense of coherence; and health status and health habits.

The questionnaires include instruments already translated and validated in Arabic as part of the CHART (Changing health and health care needs along the Syrian refugees’ trajectories to Norway) study [27], including the sociodemographic questions. The Well-Being Index WHO (Five) and the 13-item Sense of Coherence Scale (SOC-13) [28, 29] are used in the second part of the questionnaire. For the assessment of physical and mental health in the third part, health-related risk factors and use of non-prescribed medication, validated questions from The Nord-Trøndelag Health Study (HUNT 3) are used [30]. For the assessment pain, the Brief Pain Inventory (BPI) — Short Form is used [25]. To assess individual distress caused by traumatic events, the Impact of Events Scale — Revised (IES-R 22) is used [26]. The General Health Questionnaire (GHQ-12) is used to identify non-psychotic and minor psychiatric disorders in the general population and is sensitive to short-term psychiatric disorders [31].

Systematic qualitative observation following a pre-structured scheme (Additional file 3) will be conducted by a bilingual researcher (PhD candidate) sitting in the room during the intervention and taking detailed notes regarding when and how the intervention is introduced and conducted, the questions being asked, any actions taken by the team leaders to implement the effect of the interventions in situ and how participants interact with each other and with the team leader during the training session. Each group will be observed at least three times (1 h for PAAI sessions and 2.5 h for TRT sessions) with the aim of capturing changes and processes after consent from the members of the group.

To further understand the mechanisms of action and participants’ experiences as well as investigating the fit with the broader context of care delivery, we will interview participants from each group after the intervention is finished. The interviews will be recorded and transcribed verbatim. No personal data will be linked with the recorded material. Questions used will be adapted from those used by Sarkadi et al. [23] in Sweden in their evaluation of a group intervention for unaccompanied refugee minors with symptoms of PTSD.

### Outcomes

The study has two main outcome measures: pain will be measured through mean scores assessed by the BPI, with a range from 0 to 10 (the cutoff point is set as scores ≥ 3); post-traumatic symptoms will be measured through the IES-R, with a range from 0 to 88. Mean scores will be calculated. Treatment effects will be measured by comparing the differences in main outcomes assessed from the post-intervention questionnaire in the immediate group compared to the pre-intervention questionnaire in the delayed list group. As secondary outcomes, psychological disorders will be assessed through the GHQ-12 with a range from 0 to 36, for which mean scores will be calculated and differences between the immediate and delayed interventions groups adjusting for pain/IESR score at baseline will be assessed.

### Analysis and statistical methods

Baseline characteristics will be presented separately for the two groups within each of the two trial arms (PAAI and TRT), with medians and interquartile range for continuous variables and counts and percentages for categorical variables. All data will be analyzed according to the intention-to-treat principle. All tests will be two-sided and 5% will be used as the level of significance. The trial will follow the CONSORT guidelines for publication of results.

The immediate effect of the interventions will be assessed by comparing the scores of the immediate intervention participants at the last treatment session (6 or 8 weeks for TRT and the PAAI, respectively) with the scores of the delayed intervention participants right after they begin their intervention. This will mean a 1-week delay in the measurements of both groups. The effect after 6/8 weeks will be assessed using linear regression, with the continuous measure of the outcome at 6/8 weeks (BPI, IESR) as the dependent variable and the allocation group as the independent variable with adjustment for baseline score of the outcome. The effect will be reported as regression coefficients with 95% confidence intervals and can be interpreted as the mean difference in scores after 6/8 weeks between the immediate intervention and the delayed intervention groups after adjustment for potential differences in outcome at baseline. Because group dynamics and other aspects of group membership could cause correlations between individuals within groups, we will calculate intraclass correlation coefficients for the outcomes and apply mixed-effects linear regression with random intercept and slope for group membership. Missing data will be considered, and appropriate multiple imputations based on characteristics measured at baseline will be done when necessary. As all participants will receive the intervention, additional cohort analyses will be conducted to supplement evaluation of the duration of the effect of the interventions.

### Potential harms

There is a potential risk of worsening mental or pain symptoms through the interventions, and the leaders of the groups are trained to identify and treat or refer patients that present signs of worsening during the meetings. If substantial worsening is found among the participants at the last assessment in either mental or pain symptoms, individual interventions will be recommended based on advice from involved clinicians. The interpreter will be present for 30 min after each session so that there is time to debrief and deal with what happened in the group that day. This is beneficial both for safeguarding the participants and also for the interpreters, who also often have a refugee background and might be affected by the happenings in the group. There are no provisions for post-trial care other than those included in the regular health system. Norway has a patient insurance schema, which could be relevant for compensation if the participants are harmed by participation.

### Data management and monitoring

The core study team for the CHART study (Health and health care needs among Syrian refugees), of which this RTC is a part (https://www.uib.no/en/generalpractice/chart), is composed by the principal investigator of the study, three other senior researchers and three PhD students, one of them with the main responsibility for this part of the study. Indeed, coordination is a main issue in the implementation of a RCT with two arms like ours. The core study team and Bergen municipality will have regular meetings to check the overall progress of the interventions, ensure adherence to the protocol, quality of the study and ethical conduct. In addition, the Centre for Crisis Psychology has certified all health professionals and collaborating interpreters involved in the TRT intervention, and has regular contact with the municipality and the university to ensure fidelity to their intervention. Interpreters working for the PAAI will attend a course to learn the special language/terminology used during the PAAI in advance.

The University of Bergen is responsible for collecting and plotting the data. Double data entry will be conducted, and data will be stored on a secure data server. Data cleaning with range checks for data values will be conducted before analysis.

Although we have an external reference group for the CHART study composed of national and international stakeholders, including user representatives, there will not be an independent auditing or data monitoring committee.

## Discussion

In this study, we will develop and test two interventions to treat common symptoms among Syrian refugees in Norway: pain and post-traumatic symptoms. We have chosen to develop group-based interventions in collaboration with the users and the health care providers who usually deliver the services. This involves accommodating their suggestions when it comes to delivery, aiming to produce treatment options that are feasible and scalable to the rest of the refugee population if proven safe and effective. Our research project will improve the knowledge on the impact of group physiotherapy and TRT among refugees from Syria with pain disorders or post-traumatic symptoms, and this evidence will be probably applicable to other groups of refugees with minimal adaptation.

The design of the study has some limitations and several strengths. The study is individually randomized, minimizing potential confounding. The study size should be sufficient to answer the primary objectives with reasonable precision; however, recruiting refugees has previously been difficult for other studies. We have therefore invested time and effort to establish a relationship with the community based on trust, mutual benefit and feedback and have involved users from the beginning. The study includes several researchers with refugee background themselves and builds upon a main study in which recruitment has been successful [27]. However, as the trial is not blinded, this could introduce information bias or the Hawthorne effect but at the same time contributes to improved external validity [32]. Ideally, we should have used a full factorial design to be able to estimate the effect of TRT on pain, the effect of the PAAI on mental health symptoms and a possible interaction effect between TRT and the PAAI, but this would require a much larger sample size for both trials and the numbers of Syrian refugees with pain or mental health symptoms in Bergen would not be enough. Comorbidity between mental health problems and pain disorder is common. The PAAI is expected to also have some effect on mental health problems in addition to its assumed main effect on pain reduction, and TRT is also expected to have some effect on pain in addition to its assumed main effect on trauma symptom reduction. Using one intervention as a control for the other would therefore not be adequate. Denying participants access to treatment when diagnosed with symptoms would be unethical, therefore we opted for a delayed intervention. Thus, we chose an immediate versus delayed intervention study design for both interventions. The study is funded from public sources, ensuring independence.

## Trial status

The study was registered at Clinical Trials.gov on 19 February 2019 (ID: NCT03951909). Enrolment started in July 2018 and was completed in September 2019.

## Supplementary information

**Additional file 1.** Baseline Questionnaire Q0.

**Additional file 2.** Follow-up Questionnaire Q1.

**Additional file 3.** Embedded process evaluation.

**Additional file 4.** Informed consent form.

**Additional file 5.** SPIRIT 2013 Checklist: Recommended items to address in a clinical trial protocol and related documents.

## Data Availability

The datasets that will be generated and/or analyzed during the current study will not be publicly available due to confidentiality of sensitive data, but are available from the corresponding author on reasonable request and following the Norwegian ethical norms. The data will be analyzed and published in peer-reviewed journals by the study group. Authorship will follow Vancouver rules.

## Abbreviations

BPI: Brief Pain Inventory
GHQ: General Health Questionnaire
HUNT 3: The Nord-Trøndelag Health Study
IESR: Impact Event Scale — Revised
PAAI: Physiotherapy Activity and Awareness Intervention
PTSD: Post-traumatic stress disorders
Q0: Questionnaire 0
Q1: Questionnaire 1
SD: Standard deviation Assigned to TRT
SOC-13: Sense of Coherence Scale-13
TRT: Teaching Recovery Techniques
WHO (Five): Well-Being Index-5
