# Supplementary material for: Two interventions to treat pain disorders and post-traumatic symptoms among Syrian refugees: protocol for a randomized controlled trial
Source: Trials. 2019 Dec 27;20:784. doi: 10.1186/s13063-019-3919-x (PMC6935096; doi:10.1186/s13063-019-3919-x)
Supplement: Supplementary file 2 — Additional file 2. Follow-up Questionnaire Q1. [file 13063_2019_3919_MOESM2_ESM.pdf]

Date:

Place:

Effect of physiotherapy and psychological group treatment on physical and mental health among refugees from Syria with pain disorders or post-traumatic symptoms

QUESTIONNAIRE Q1

Thank you for taking part in this study!

The information in this questionnaire will be used in research aimed to understand the effect of treatment in your health situation and to improve health care services for refugees. It is important that you answer all the questions. Please ask if there is something you do not understand. The completed questionnaire should be given back to the person who invited you to the study before you leave.

Please answer by putting an X in the box (☐), or answering the open fields ( ) as explained in the text.

By answering this questionnaire you accept that we use this information only for the purpose explained to you. All information will be treated in strict confidence.

Yours sincerely,
University of Bergen and Health Care services at the Municipality of Bergen.

FOR THE FIELD WORKER:

Has the participant already answered another questionnaire for this project (make sure that he/she knows which project you are talking about)? Has the person already participated in the Syria-health study?

No	Yes, in Bergen	Yes, in Kristiansand	Yes, in Lebanon	Yes, elsewhere
☐	☐	☐	☐	☐

HEALTH LITERACY SCREENING

1 **How often do you need to have someone help you when you read instructions, pamphlets, or other written material from your doctor or pharmacy?**

Never	Rarely	Sometimes	Often	Always
☐	☐	☐	☐	☐

PART 1 – BACKGROUND INFORMATION

2 **Name:**

Please specify.

3 **Mobile phone number:**

Please specify (e.g. 123 45 678).

4 **Date of birth:** __ . __ . ____ *(e.g. 01.06.1978)*

5 **What is your status in Norway now?**

☐ Asylum seeker ☐ Refugee ☐ Other

Survey number/ID:

PART 2 – WELL-BEING

6 Please indicate for each of the five statements which is closest to how you have been feeling over the last two weeks. Notice that higher numbers mean better well-being.

Example: If you have felt cheerful and in good spirits more than half of the time during the last two weeks, put a tick in the box with the number 3 in the upper right corner.

	All of the time					At no time
6.1 I have felt cheerful and in good spirits	5	4	3	2	1	0
6.2 I have felt calm and relaxed	5	4	3	2	1	0
6.3 I have felt active and vigorous	5	4	3	2	1	0
6.4 I woke up feeling fresh and rested	5	4	3	2	1	0
6.5 My daily life has been filled with things that interest me	5	4	3	2	1	0

7 Here is a series of questions relating to various aspects of your life. Each question has seven possible answers. Please mark the number, which expresses your answer, with number 1 and 7 being the extreme answers. If the words under 1 are right for you, circle 1: if the words under 7 are right for you, circle 7. If you feel differently, circle the number which best expresses your feeling. Please give only one answer to each question.

	Very seldom or never						Very often
7.1 Do you have the feeling that you don't really care about what goes on around you?	1	2	3	4	5	6	7

	Never happened						Always happened
7.2 Has it happened in the past that you were surprised by the behaviour of people whom you thought you knew well?	1	2	3	4	5	6	7

	Never happened						Always happened
7.3 Has it happened that people whom you counted on disappointed you?	1	2	3	4	5	6	7

	No clear goals or purpose at all						Very clear goals and purpose
7.4 Until now your life has had:	1	2	3	4	5	6	7

	Very often						Very seldom or never
7.5 Do you have the feeling that you're being treated unfairly?	1	2	3	4	5	6	7

	Very often						Very seldom or never
7.6 Do you have the feeling that you are in an unfamiliar situation and don't know what to do?	1	2	3	4	5	6	7

	A source of deep pleasure and satisfaction						A source of pain and boredom
7.7 Doing the thing you do every day is:	1	2	3	4	5	6	7

	Very often						Very seldom or never
7.8 Do you have very mixed-up feelings and ideas?	1	2	3	4	5	6	7

	Very often						Very seldom or never
7.9 Does it happen that you have feelings inside you would rather not feel?	1	2	3	4	5	6	7

Survey number/ID:

	Never						Very often
7.10 Many people – even those with a strong character – sometimes feel like sad sacks (losers) in certain situations. How often have you felt this way in the past?	1	2	3	4	5	6	7

	You overestimated or underestimated its importance						You saw things in the right proportion
7.11 When something happened, have you generally found that:	1	2	3	4	5	6	7

	Very often						Very seldom or never
7.12 How often do you have the feeling that there's little meaning in the things you do in your daily life?	1	2	3	4	5	6	7

	Very often						Very seldom
7.13 How often do you have feelings that you're not sure you can keep under control?	1	2	3	4	5	6	7

PART 3 – HEALTH STATUS AND HEALTH HABITS

8 How do you consider your health at the moment?

Very poor	Poor	Neither	Good	Very good
☐	☐	☐	☐	☐

9 Have you used any of the following medicines?
(Please place only one X for each medication at the answer that best fits your situation.)

	Daily	Weekly	Less than weekly	Never used
9.1 Painkillers, off prescription	☐	☐	☐	☐
9.2 Painkillers, on prescription	☐	☐	☐	☐
9.3 Sedatives	☐	☐	☐	☐
9.4 Tranquillizers	☐	☐	☐	☐
9.5 Anti-depressive medication	☐	☐	☐	☐
9.6 Other prescribed medication, but do not know for what	☐	☐	☐	☐

10 How often do you exercise?
(On average. Put an X in only one box)

- ☐ Never
- ☐ Less than once a week
- ☐ Once a week
- ☐ 2-3 times a week
- ☐ Nearly every day

11 About how many hours do you sit during a normal day?
(Both work hours and leisure time)

About [|] hours *(e.g. 6 hours)*

	Yes	No
**12 Do you have physical pain now that has lasted more than 6 months?**	☐	☐

13 If yes, how strong has your physical pain been during the last 4 weeks?

No pain	Very mild	Mild	Moderate	Strong	Very strong
☐	☐	☐	☐	☐	☐

BRIEF PAIN INVENTORY (SHORT FORM)

	Yes	No
**14 Throughout our lives, most of us have had pain from time to time (such as minor headaches, sprains, and toothaches). Have you had pain other than these everyday kinds of pain today?**	☐	☐

15 On the diagram, shade in the areas where you feel pain. Put an X on the area that hurts the most.

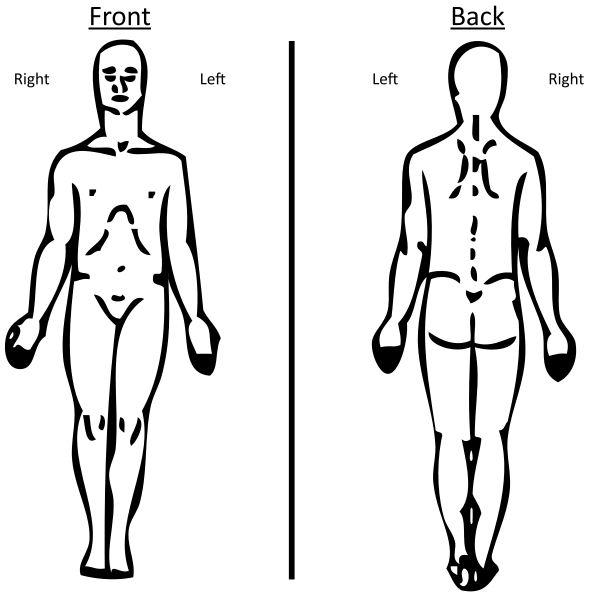

Survey number/ID:

16 **Please rate your pain by marking the box beside the number that best describes your pain at its worst in the last 24 hours.**

No pain									Pain as bad as you can imagine
1	2	3	4	5	6	7	8	9	10

17 **Please rate your pain by marking the box beside the number that best describes your pain at its least in the last 24 hours.**

No pain									Pain as bad as you can imagine
1	2	3	4	5	6	7	8	9	10

18 **Please rate your pain by marking the box beside the number that best describes your pain on the average.**

No pain									Pain as bad as you can imagine
1	2	3	4	5	6	7	8	9	10

19 **Please rate your pain by marking the box beside the number that tells how much pain you have right now.**

No pain									Pain as bad as you can imagine
1	2	3	4	5	6	7	8	9	10

20 **What treatments or medications are you receiving for your pain?**

Please specify.

21 **In the last 24 hours, how much relief have pain treatments or medications provided? Please mark the box below the percentage that most shows how much relief you have received.**

No relief									Complete relief
10%	20%	30%	40%	50%	60%	70%	80%	90%	100%

22 **Mark the box beside the number that describes how, during the past 24 hours, pain has interfered with your:**

22.1 **General activity**

Does not interfere									Completely interferes
1	2	3	4	5	6	7	8	9	10

22.2 **Mood**

Does not interfere									Completely interferes
1	2	3	4	5	6	7	8	9	10

22.3 **Walking ability**

Does not interfere									Completely interferes
1	2	3	4	5	6	7	8	9	10

22.4 **Normal work**
(includes both work outside the home and housework)

Does not interfere									Completely interferes
1	2	3	4	5	6	7	8	9	10

22.5 **Relations with other people**

Does not interfere									Completely interferes
1	2	3	4	5	6	7	8	9	10

22.6 **Sleep**

Does not interfere									Completely interferes
1	2	3	4	5	6	7	8	9	10

22.7 **Enjoyment of life**

Does not interfere									Completely interferes
1	2	3	4	5	6	7	8	9	10

23 **Exposure to a stressful event or situation (either short or long lasting) of exceptionally threatening or catastrophic nature is likely to cause pervasive distress in almost anyone. Examples of such difficult and frightening experiences are: being assaulted, or witnessing other people being hurt or killed.**

	Yes	No
Have you experienced any of these or some other terrifying event(s)?	☐	☐

Survey number/ID:

IMPACT OF EVENTS SCALE - REVISED (IES-R)

INSTRUCTIONS: Below is a list of difficulties people sometimes have after stressful life events. Please read each item, and then indicate how distressing each difficulty has been for you **during the past seven days** with respect to ____________ (event) that occured on ________ (date). How much have you been distressed or bothered by these difficulties?

	Not at all	A little bit	Moderately	Quite a bit	Extremely
24.1 Any reminder brought back feelings about it.	0	1	2	3	4
24.2 I had trouble staying asleep.	0	1	2	3	4
24.3 Other things kept making me think about it.	0	1	2	3	4
24.4 I felt irritable and angry.	0	1	2	3	4
24.5 I avoided letting myself get upset when I thought about it or was reminded of it.	0	1	2	3	4
24.6 I thought about it when I didn't mean to.	0	1	2	3	4
24.7 I felt as if it hadn't happened or wasn't real.	0	1	2	3	4
24.8 I stayed away from reminders of it.	0	1	2	3	4
24.9 Pictures about it popped into my mind.	0	1	2	3	4
24.10 I was jumpy and easily startled.	0	1	2	3	4
24.11 I tried not to think about it.	0	1	2	3	4
24.12 I was aware that I still had a lot of feelings about it but I didn't deal with them.	0	1	2	3	4
24.13 My feelings about it were kind of numb.	0	1	2	3	4
24.14 I found myself acting or feeling like I was back at that time.	0	1	2	3	4
24.15 I had trouble falling asleep.	0	1	2	3	4
24.16 I had waves of strong feelings about it.	0	1	2	3	4
24.17 I tried to remove it from my memory.	0	1	2	3	4
24.18 I had trouble concentrating.	0	1	2	3	4
24.19 Reminders of it caused me to have physical reactions, such as sweating, trouble breathing, nausea, or a pounding heart.	0	1	2	3	4
24.20 I had dreams about it.	0	1	2	3	4
24.21 I felt watchful and on-guard.	0	1	2	3	4
24.22 I tried not to talk about it.	0	1	2	3	4

Survey number/ID:

GHQ-12

25 **We would like to know how you have been feeling the last couple of months. Please mark the option that best suits your situation.**

During the last two weeks, have you:

25.1 Been able to concentrate on what you're doing?	Better than usual	As usual	Less than usual	A lot less than usual
25.2 Lost much sleep over worry?	Has not happened	Not more than usual	More than usual	I slept a lot less than usual
25.3 Felt that you are playing a useful part in things?	More than usual	As usual	Less than usual	A lot less than usual
25.4 Felt capable of making decisions about things?	More than usual	As usual	Less than usual	A lot less than usual
25.5 Felt constantly under strain?	Not at all	Not more than usual	More than usual	A lot more than usual
25.6 Felt you couldn't overcome your difficulties?	Not at all	Not more than usual	More than usual	A lot more than usual
25.7 Been able to enjoy your normal day to day activities?	More than usual	As usual	Less than usual	A lot less than usual
25.8 Been able to face up to your problems?	Better than usual	As usual	Less than usual	A lot less than usual
25.9 Been feeling unhappy or depressed?	Not at all	Not more than usual	More than usual	A lot more than usual
25.10 Been losing confidence in yourself?	Not at all	Not more than usual	More than usual	A lot more than usual
25.11 Been thinking of yourself as a worthless person?	Not at all	Not more than usual	More than usual	A lot more than usual
25.12 Been feeling reasonably happy for day-to-day activities?	More than usual	As usual	Less than usual	A lot less than usual

THANK YOU FOR ANSWERING THESE QUESTIONS! PLEASE MAKE SURE TO RETURN THIS FORM TO THE PERSON WHO GAVE IT TO YOU BEFORE LEAVING.

Survey number/ID:
