# Supplementary material for: Two interventions to treat pain disorders and post-traumatic symptoms among Syrian refugees: protocol for a randomized controlled trial
Source: Trials. 2019 Dec 27;20:784. doi: 10.1186/s13063-019-3919-x (PMC6935096; doi:10.1186/s13063-019-3919-x)
Supplement: Supplementary file 3 — Additional file 3. Embedded process evaluation. [file 13063_2019_3919_MOESM3_ESM.pdf]

Embedded process evaluation

Evaluation scheme to be used in interventions (3 times / per group)

Reminder to the group that the researcher is only there to note and not to interpret or answer questions while the group is in progress. Try to be as "invisible" as possible in the room.

1- How is the intervention presented/implemented?

- Interpreter: Present? Sex? Dialect?
- Questions from participants to the leader of the group — what has been asked? Note specific questions (not need answers)
- Misunderstandings?
- Do instructors change any part of the intervention? If something, what?
- Participants' body language? E.g. mainly sitting in open or "closed" positions (hands in cross etc. subject to cultural differences)
- Are participants? (Give examples)
    - All active alike?
    - Passive?
    - Asking many questions?
    - Satisfied?
    - Encouraged by instructors to activity?
    - Participants contact the instructors via another participant

2- How do participants interact? (Nice, social, laughter, tense, supportive)

3- Language (Arabic, Kurdish, Norwegian)

4- Heterogeneity and homogeneity in the group: Are there similarities and/or differences between group members: clothing, roles etc.

5- Other relevant points noticed.

Interpreter:

Instructor:

Group:

Date:
