# Supplementary material for: Two interventions to treat pain disorders and post-traumatic symptoms among Syrian refugees: protocol for a randomized controlled trial
Source: Trials. 2019 Dec 27;20:784. doi: 10.1186/s13063-019-3919-x (PMC6935096; doi:10.1186/s13063-019-3919-x)
Supplement: Supplementary file 4 — Additional file 4. Informed consent form. [file 13063_2019_3919_MOESM4_ESM.docx]

**Informed consent form-questionnaire**

***‘*Effect of physiotherapy and psychological group treatment on physical and mental health among refugees from Syria with pain disorders or post-traumatic symptoms”**

**Background information:**

The University of Bergen together with the municipalities of Bergen are conducting a study about the effect of two interventions on the health of Syrian refugees and asylum seekers to Norway. The results from the study will increase our knowledge about the health of refugees in Norway and will help us to provide better health care services. This is an invitation for you to participate in this study by answering a questionnaire survey.

**Participant selection:**

All 16 years or older persons from Syria with pain disorders of post-traumatic stress symtoms are invited to participate in this study by answering a self-administered anonymous questionnaire. The questionnaire takes 20-25 minutes to complete, and includes general demographic questions, questions related to health, well-being, health status and health habits.

**Confidentiality:**

We will register your name and personal identification in information in the questionnaire only as long as you are part of the study and in order to contact you for the interventions. All personal information will be deleted after the study. Information about you that will be collected through the questionnaire will be kept confidential and stored safely. Only the researchers will have access to your information, which will only be used in accordance with the purpose of the study as described above. This study is not linked to any other legal institution and cannot affect your eventual permission or denial to stay in the country.

**Rights to refuse or withdraw:**

Participating in the Syrian Refugee Health Survey is your choice. You do not have to take part in this research if you do not wish to do so and refusing to participate will not affect your regular health exam or treatment in any way. You may stop participating in the research at any time that you wish without losing any of your rights as a patient here. If you have any questions about this survey, please talk to the person who gave you the questionnaire.

**Consent for participation in the study:**

I have read the foregoing information, or it has been read to me. I have had the opportunity to ask questions about it and any questions that I have asked have been answered to my satisfaction. I consent voluntarily to participate as a participant in this research. I know that I may refuse to participate or to stop at any time without any loss of health care benefits that I am otherwise receiving.

Date

Respondent Signature Interviewer Signature
